# Myocardial performance in conscious streptozotocin diabetic rats

**DOI:** 10.1186/1475-2840-5-26

**Published:** 2006-12-04

**Authors:** Giulianna R Borges, Mauro de Oliveira, Helio C Salgado, Rubens Fazan

**Affiliations:** 1 Department of Physiology, School of Medicine of Ribeirão Preto, University of São Paulo, Ribeirão Preto, SP, 14049-900, Brazil.

## Abstract

**Background:**

In spite of a large amount of studies in anesthetized animals, isolated hearts, and in vitro cardiomyocytes, to our knowledge, myocardial function was never studied in conscious diabetic rats. Myocardial performance and the response to stress caused by dobutamine were examined in conscious rats, fifteen days after the onset of diabetes caused by streptozotocin (STZ). The protective effect of insulin was also investigated in STZ-diabetic rats.

**Methods:**

Cardiac contractility and relaxation were evaluated by means of maximum positive (+dP/dt_max_) and negative (-dP/dt_max_) values of first derivative of left ventricular pressure over time. In addition, it was examined the myocardial response to stress caused by two dosages (1 and 15 μg/kg) of dobutamine. One-way analysis of variance (ANOVA) was used to compare differences among groups, and two-way ANOVA for repeated measure, followed by Tukey post hoc test, to compare the responses to dobutamine. Differences were considered significant if P < 0.05.

**Results:**

Basal mean arterial pressure, heart rate, +dP/dt_max _and -dP/dt_max _were found decreased in STZ-diabetic rats, but unaltered in control rats treated with vehicle and STZ-diabetic rats treated with insulin. Therefore, insulin prevented the hemodynamic and myocardial function alterations observed in STZ-diabetic rats. Lower dosage of dobutamine increased heart rate, +dP/dt_max _and -dP/dt_max _only in STZ-diabetic rats, while the higher dosage promoted greater, but similar, responses in the three groups. In conclusion, the results indicate that myocardial function was remarkably attenuated in conscious STZ-diabetic rats. In addition, the lower dosage of dobutamine uncovered a greater responsiveness of the myocardium of STZ-diabetic rats. Insulin preserved myocardial function and the integrity of the response to dobutamine of STZ-diabetic rats.

**Conclusion:**

The present study provides new data from conscious rats showing that the cardiomyopathy of this pathophysiological condition was expressed by low indices of contractility and relaxation. In addition, it was also demonstrated that these pathophysiological features were prevented by the treatment with insulin.

## Background

Besides increased incidence of autonomic neuropathy and coronary artery disease, diabetes has been considered an independent risk for myocardial dysfunction [1,2].

It is well documented in experimental diabetes that both left ventricular diastolic [3,4] and systolic dysfunction [5,6] are attributed to diabetic cardiomyopathy, as usually seen under clinical condition [1,3].

A deleterious effect of diabetes on cardiac performance has been well documented in isolated heart preparation [7], isolated cardiomyocytes [5] and intact anesthetized animals [8]. Recently, Yoon et al. [7] showed in chronic diabetic rats, under anesthesia, a decrease of both myocardial contractility and relaxation. However, in spite of a large amount of studies in anesthetized animals, isolated hearts, and in vitro cardiomyocytes, to our knowledge, myocardial function was never studied in conscious diabetic rats. Besides myocardial dysfunction, it is well known that experimental diabetes affects hemodynamic parameters such as heart rate and arterial pressure [9,10].

Myocardial stress produced by dobutamine is a useful tool for evaluating cardiac events in humans [11] and experimental animals [8]. This β_1_-adrenergic receptor agonist promotes an increase in both contractility and heart rate. Therefore, dobutamine was used to evaluate the degree of attenuation of the inotropism, lusitropism and chronotropism exhibited by STZ-diabetic rats.

It is well documented that insulin can prevent, or revert, a number of outcomes caused by experimental diabetes [12,13]. In fact, insulin normalizes not only blood glucose levels [12], but most of the metabolic parameters of diabetic rats [14,15]. Studies from isolated papillary muscle [12] and isolated heart preparations [16] showed that insulin reversed, or prevented, contractile dysfunction in diabetic rats. Therefore, a protocol was carried out to examine the protective effect of this hormone on the hemodynamic and myocardial function of STZ-diabetic rats.

In the present study, STZ was used to produce experimental diabetes, such that myocardial contractility and relaxation was examined in conscious rats. From the methodological point of view cardiac function was indirectly assessed by means of the first derivative of left ventricular pressure over time, more specifically, the maximum rate of isovolumic pressure development (+dP/dt_max_, an inotropic index) and the maximum rate of isovolumic pressure decay (-dP/dt_max_, a lusitropic index). Hemodynamic parameters, such as heart rate and arterial pressure were evaluated as well. Moreover, myocardial stress test with dobutamine was used to evaluate the degree of attenuation of myocardial performance of STZ-diabetic rats. Finally, a protocol was carried out to examine the protective effect of insulin on the hemodynamic and myocardial function of STZ-diabetic rats.

## Methods

### Experimental Diabetic Model

Male Wistar rats (~300 g) received a single injection of STZ (50 mg/kg, dissolved in 0.01 M citrate buffer, pH 4.5) into the penile vein, after a fasting overnight for approximately 12 hours. An equivalent volume (0.1 mL) of vehicle (0.01 M citrate buffer, pH 4.5) was administered to control rats. Development of diabetes was confirmed 72 h later by the presence of hyperglycemia (> 350 mg/dL). Half of STZ-diabetic rats was selected to receive daily injection of insulin NPH (9 UI/kg/day, s.c.), starting 3 days after STZ injection. Blood glucose measurement (Beckman Glucose Analyzer, Beckman Instruments Inc., Fullerton, EUA) was performed 3 and 15 days after STZ or vehicle administration. The three groups were studied 15 days after the administration of STZ (n = 9), or vehicle (n = 8), or STZ associated with insulin (n = 8).

### Surgical Protocol

The animals were anesthetized with tribromoethanol (250 mg/kg, i.p.) and the right common carotid artery was isolated and indwelled with a PE50 polyethylene catheter, whose tip was inserted into the left ventricle. Two other polyethylene catheters (PE10 soldered to PE50) were inserted into the femoral artery and vein for arterial pressure recording and drug administration, respectively. The catheters were exteriorized in the back of the neck. After the surgical procedure the rats were placed in individual cages for surgical recovery, during approximately 24 h, with free access to food and tap water.

### Blood Pressure and Left Ventricular Pressure Recording

On the day of the experiment, without the effect of anesthesia, the animals were brought to the experimental room with at least 30 min in advance, in order to adapt to the laboratory. The arterial and ventricular catheters were connected to pressure transducers (P23Gb, Statham, Hato Hey, PR) and the signals were amplified (CL-615422-1, Gould Instruments Systems, Inc., Valley View, USA) and digitally sampled (1 kHz) on an IBM/PC equipped with an analog to digital interface (DI-220, Dataq Instruments, Akron, USA). During the recordings the rats were allowed to move, freely, inside the cage.

### Experimental Protocol

After 5 min of basal recording of arterial and left ventricular pressure, 1 and 15 μg/kg of dobutamine were administered intravenously in a random sequence. Ten to 15 min elapsed between each dosage.

All procedures used in the present study adhered to The Guide for the Care and Use of Laboratory Animals prepared by the National Academy of Sciences and published by the National Institute of Health (NIH Publication n° 80-23, revised 1978), and the experimental protocols used in this research were reviewed and approved by the Committee of Ethics in Animal Research of the School of Medicine of Ribeirão Preto, University of São Paulo.

### Data Analysis

Pulsatile AP recordings were processed by a computer software (Advanced CODAS/Windaq, Dataq Instruments, Akron, USA) that applies an algorithm to detect inflection points of a periodic waveform determining, beat-by-beat, values of systolic and diastolic pressures. Heart rate was calculated every cardiac cycle from the interval between successive values of diastolic arterial pressure. The first derivative of left ventricular pressure was calculated, and the maximum rate of isovolumic pressure development (+dP/dt_max_) and the maximum rate of isovolumic pressure decay (-dP/dt_max_) values were used as indices of contractility and relaxation, respectively. The response of the parameters investigated, arterial pressure, heart rate, +dP/dt_max _and -dP/dt_max_, to dobutamine, were calculated by the difference between the maximal response and the baseline value immediately before dobutamine administration.

### Statistical Analysis

Data are reported as mean ± SEM. One-way analysis of variance (ANOVA) was used to compare differences among groups, and two-way ANOVA for repeated measure, followed by Tukey post hoc test, to compare the responses to dobutamine. Differences were considered significant if P < 0.05.

## Results

### Weight and Blood Glucose

Table 1 shows weight and blood glucose levels of the three groups studied. STZ-diabetic rats developed severe hyperglycemia associated with decreased body weight. On the other hand, control rats and STZ-diabetic rats treated with insulin exhibited normal weight gain and blood glucose levels.

**Table 1:** Weight and blood glucose levels.

	**Weight**			**Blood Glucose**	
	**1^**st **^day**	**3^**rd **^day**	**15^**th **^day**	**3^**rd **^day**	**15^**th **^day**
	
**Control**	323 ± 10	344 ± 8	446 ± 9^‡^	107 ± 3	109 ± 3
**STZ**	348 ± 13	330 ± 15	296 ± 6^†§^	453 ± 18*	444 ± 17^§^
**STZ + Insulin**	303 ± 15	335 ± 3	416 ± 11^‡^	464 ± 35*	101 ± 3

Weight (g) and blood glucose (mg/dL) of control, STZ-diabetic and STZ-diabetic rats treated with insulin (STZ+Insulin). Data are reported as mean ± SEM. *P < 0.001 as compared to control group; ^†^P < 0.05 as compared to 1^st ^day; ^‡^P < 0.001 as compared to 1^st ^and 3^rd ^day; ^§^P < 0.001 as compared to control and STZ+Insulin group.

### Hemodynamic and Cardiac Function Parameters

Table 2 shows baseline values of mean arterial pressure, heart rate, +dP/dt_max _and -dP/dt_max _of the three groups studied. STZ-diabetic rats show significant reduction of these parameters, while control rats and STZ-diabetic rats treated with insulin did not exhibit any alteration of these parameters.

**Table 2:** Basal hemodynamic parameters

	**MAP**	**HR**	**+dP/dt_**max**_**	**-dP/dt_**max**_**
	
**Control**	103 ± 3	359 ± 5	12676 ± 723	-8207 ± 339
**STZ**	91 ± 3*	312 ± 7*	9512 ± 437*	-5763 ± 422*
**STZ + Insulin**	105 ± 4	357 ± 11	13943 ± 863	-8985 ± 315

Mean arterial pressure (MAP; mmHg), heart rate (HR bpm), maximum rate of isovolumic pressure development (+dP/dt_max_, mmHg/s) and maximum rate of isovolumic pressure decay (-dP/dt_max_, mmHg/s) of control, STZ-diabetic and STZ-diabetic rats treated with insulin (STZ+Insulin). Data are reported as mean ± SEM. * P < 0.05 as compared to control and STZ+Insulin group.

### β_1_-Adrenergic Receptor Stimulation with Dobutamine

**Figure 1:**
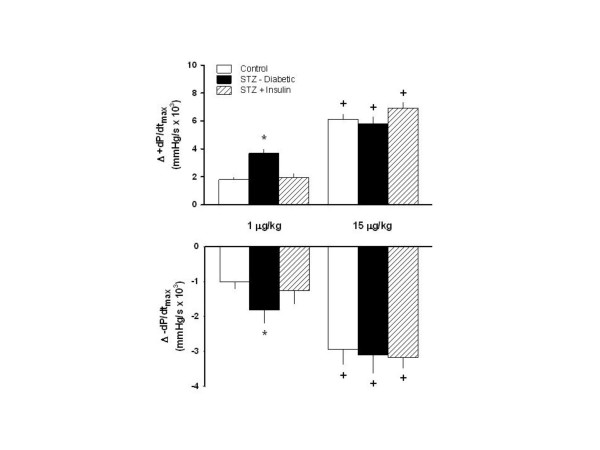
Responses of +dP/dt_max _and -dP/dt_max _to β_1_-adrenergic stimulation with dobutamine (1 and 15 μg/kg) in conscious control (treated with vehicle) rats, and conscious diabetic rats obtained by means of streptozotocin (STZ), treated (STZ+Insulin), or not (STZ-diabetic), with insulin. Data are reported as means ± SEM. *P < 0.05 compared to the other groups stimulated with 1 μg/kg; ^+^P < 0.05 compared to their counterparts stimulated with 1 μg/kg.

The responses of +dP/dt_max _and -dP/dt_max _to dobutamine in the three groups studied are shown in Figure 1. STZ-diabetic rats exhibited a greater response of +dP/dt_max _and -dP/dt_max _to 1 μg/kg of dobutamine as compared to control rats, while insulin prevented this greater response. The dosage of 15 μg/kg of dobutamine elicited a higher dP/dt response in the three groups, as compared to the response caused by 1 μg/kg, even though the response to 15 μg/kg of dobutamine did not differ among the three groups. Insulin did not affect the enhanced response to the higher dosage of dobutamine, i.e 15 μg/kg of dobutamine.

**Figure 2:**
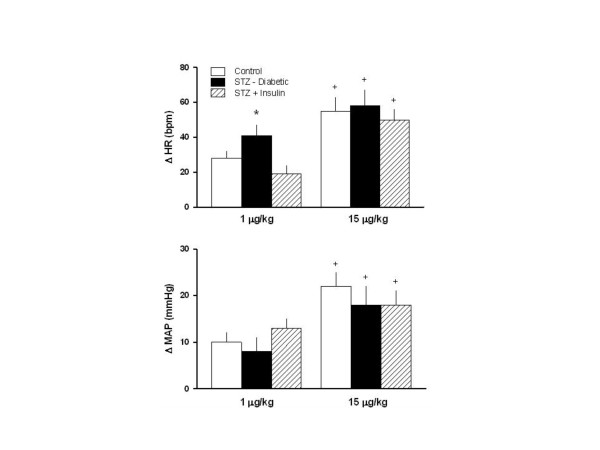
Responses of heart rate and mean arterial pressure to β_1_-adrenergic stimulation with dobutamine (1 and 15 μg/kg) in conscious control (treated with vehicle) rats, and conscious diabetic rats obtained by means of streptozotocin (STZ), treated (STZ+Insulin), or not (STZ-diabetic), with insulin. Data are reported as means ± SEM. *P < 0.05 compared to the other groups stimulated with 1 μg/kg; ^+^P < 0.05 compared to their counterparts stimulated with 1 μg/kg.

The responses of heart rate and mean arterial pressure to dobutamine, for the three groups, are shown in Figure 2. Notice that 1 μg/kg of dobutamine elicited a greater increase of heart rate in STZ-diabetic rats, while insulin prevented this greater response. Figure 2 still shows similar increase of mean arterial pressure in the three groups, without effect of insulin. On the other hand, 15 μg/kg elicited a greater response of heart rate and mean arterial pressure as compared to the responses of 1 μg/kg. However, the amplified response of heart rate and mean arterial pressure to 15 μg/kg did not differ among the three groups, and insulin did not affect the amplified response to this higher dosage of dobutamine.

## Discussion

### Body weight, blood glucose and basal hemodynamic parameters

STZ-diabetic rats presented significant hyperglycemia and low body weight gain as compared to control rats. Treatment with insulin normalized blood glucose levels and body weight gain. These findings are in agreement with the literature which indicates that insulin normalizes the metabolic control of diabetic rats [13,15].

From the hemodynamic point of view STZ-diabetic rats presented significant hypotension and bradycardia which are also in line with previous findings from the literature [9,10,17-20]. It is well documented that this resting bradycardia is associated with cardiac sympathetic nerve impairment [9,10,13]. However, studies from isolated heart preparations have indicated that STZ-induced diabetes might be associated with decreased intrinsic heart rate as well [17,21]. Hypotension has been ubiquitously described in STZ-diabetic rats [9,10,13,19,20] and the following mechanisms are postulated as responsible for this hemodynamic outcome: 1) decreased cardiac output [5]; 2) hypovolemia due to osmotic diuresis [23]; 3) impairment of sympathetic innervation of heart and vessels [10,21].

### Myocardial Contractility and Relaxation

Despite a number of studies either in anesthetized animals or in vitro preparations, to our knowledge, the present study is the first to describe in conscious STZ-diabetic rats, both, attenuated basal inotropism (+dP/dt_max_), as well as attenuated basal lusitropism (-dP/dt_max_). Myocardial dysfunction is an important feature that might be associated with a number of intrinsic alterations of cardiac myocytes [5]. There are several studies *in vivo *(anesthetized animals) and *in vitro *(Langhendorff and isolated myocytes) showing an impairment on Ca^++ ^homeostasis and Ca^++^signaling in diabetes [5,6,23]. The literature reports studies of myocardial contractility in diabetes showing conspicuous diastolic dysfunction [2,24]. Fein et al [2] described substantial deleterious effects of diabetes in cardiac papillary muscles of rats. The most significant abnormalities involved delay of the relaxation process, slow relaxation ratio and delay in peak ratio of isometric and isotonic relaxation. Nevertheless, recent studies demonstrated reduced expression of sarcoplasmic reticulum calcium-ATPase and sodium-calcium exchanger [6,23]. Decreased basal contractility in the STZ-diabetic rats, as demonstrated by means of low +dP/dt_max_, was also observed in the present study. Reports in the literature have demonstrated myocardial dysfunction in spontaneously diabetic rats [5]. It has been suggested that this myocardial derangement is typical of the cardiac myocyte of the diabetic rat, which shows significant reduction of cell shortening and attenuation of the velocities of cell shortening and relaxation, associated with low levels of cytosolic Ca^++^. The decrease in inward Ca^++ ^current, which triggers the ryanodine Ca^++ ^channel to release Ca^++ ^[6,25] from the sarcoplasmic reticulum, could be also an important factor to explain the decrease of +dP/dt_max_. Furthermore, it was reported that diabetes reduces ryanodine sensitive Ca^++ ^channel expression of cardiac myocytes in rats [6,23]. Even though the methodological approach used in the present study does not allow to assess the mechanism responsible for the alterations of myocardial function in conscious STZ-diabetic rats, the literature shows a number of studies dealing with impairment of either Ca^++ ^signaling or sarcoplasmic reticulum function [5,26], which might explain, at least partially, the findings of the present study.

An impairment of sympathetic innervation of the heart, frequently observed in diabetes [10,19,21], should be also taken into consideration in the impairment of myocardial contractility found in STZ-diabetic rats.

In addition, in the present study, treatment with insulin prevented the occurrence of alterations caused by diabetes, i.e. bradycardia, hypotension and low +dP/dt_max _and low -dP/dt_max_. Several studies demonstrated that insulin can prevent, or even reverse, the derangements caused by chronic diabetes [12,13]. Nevertheless, the mechanism responsible for this protective effect is still unknown, because diabetes is a long-standing metabolic disorder with several outcomes. It has been demonstrated in normal cardiac myocytes that insulin speeds the glucose transport into the cell [27]. However, it has been demonstrated also that insulin promotes a positive inotropic effect independent of glucose uptake [28]. According to Stroedter et al. [15] the improvement of cardiac performance caused by subcutaneous administration of insulin, or by intraportal islet transplantation, follows the normalization of cardiac metabolism. This finding suggests that the dysfunction of the heart observed in diabetes may be caused by conspicuous alterations of myocardial metabolism caused by insulin deficiency, which can be reversed by means of exogenous replacement of the hormone.

### β_1_-Adrenergic Receptor Stimulation with Dobutamine

Two dosages of dobutamine, i.e. 1 and 15 μg/kg were used to promote pharmacological stimulation of myocardial contractility, relaxation and heart rate without the effect of anesthesia. STZ-diabetic rats exhibited greater inotropic, lusitropic as well tachycardic response to the lower dosage (1.0 μg/kg) of β_1_-adrenergic receptor agonist as compared to control rats and STZ-diabetic rats treated with insulin. These findings might be explained, at least partially, by means of an up-regulation of β_1_-adrenergic receptors exhibited by STZ-diabetic rats, even though this hypothesis deserves better investigation. Previous study from our laboratory has demonstrated that even short term (5 days) STZ-diabetes produces attenuation of the sympathetic drive [10]. In addition, it has been already described that sympathetic impairment [9,20] during the development of diabetes, leads to an increase in the number of β_1_-adrenergic receptors [29,30]. However, this up-regulation seems to be of short duration, because there is a body of evidence showing a decrease in the number of β_1_-adrenergic receptor is the predominant finding in chronic diabetes [19,31,32]. On the other hand, there was no difference among the three groups concerning the responses of heart rate, +dP/dt_max _and -dP/dt_max_. to the higher dosage (15 μg/kg) of dobutamine, indicating that this dosage was not able to discriminate differences of cardiac function in control rats versus STZ-diabetic rats treated, or not, with insulin, probably due to a saturation of β_1_-adrenergic receptors [33].

## Conclusion

The findings of the present study are in line with previous reports from the literature [9,10,19,20], showing that STZ-diabetes is characterized by bradycardia and hypotension. However, the present study provides new data from conscious rats showing that the cardiomyopathy of this pathophysiological condition was expressed by low indices of contractility and relaxation. In addition, it was also demonstrated that these pathophysiological features were prevented by the treatment with insulin. Furthermore, the greater inotropic, lusitropic and tachycardic effect caused by low dosage (1 μg/kg) of dobutamine in STZ-diabetic rats might be explained by an up-regulation of β_1_-adrenergic receptors.

## Abbreviations

STZ: streptozotocin;

+dT/dt_max_: maximum positive values of first derivative of left ventricular pressure over time;

-dP/dt_max_: maximum negative values of first derivative of left ventricular pressure over time;

MAP: mean arterial pressure;

HR: heart rate;

LVP: left ventricular pressure;

LV dP/dt: first derivative of left ventricular pressure over time;

## Competing interests

The author(s) declare that they have no competing interests.

## Authors' contributions

All authors have equally contributed in the conception and drafting of the manuscript.

## References

[B1] Kannel WB, Hjortland M, Castelli WP (1974). Role of diabetes in congestive heart failure: the Framingham study. Am J Cardiol.

[B2] Fein FS, Kornstein LB, Strobeck JE, Capasso JM, Sonnenblick EH (1980). Altered myocardial mechanics in diabetic rats. Circ Res.

[B3] Regan TJ, Lyons MM, Ahmed SS, Levinson GE, Oldewurtel HA, Ahmad MR, Haider B (1977). Evidence for cardiomyopathy in familial diabetes mellitus. J Clin Invest.

[B4] Penpargkul S, Fein F, Sonnenblick EH, Scheuer J (1981). Depressed cardiac sarcoplasmic reticular function from diabetic rats. J Mol Cell Cardiol.

[B5] Ren J, Bode AM (2000). Altered cardiac excitation-contraction coupling in ventricular myocytes from spontaneously diabetic BB rats. Am J Physiol Heart Circ Physiol.

[B6] Choi KM, Zhong Y, Hoit BD, Grupp IL, Hahn H, Dilly KW, Guatimosim S, Lederer WJ, Matlib MA (2002). Defective intracellular Ca(2+) signaling contributes to cardiomyopathy in Type 1 diabetic rats. Am J Physiol Heart Circ Physiol.

[B7] Yoon YS, Uchida S, Masuo O, Cejna M, Park JS, Gwon HC, Kirchmair R, Bahlman F, Walter D, Curry C, Hanley A, Isner JM, Losordo DW (2005). Progressive attenuation of myocardial vascular endothelial growth factor expression is a seminal event in diabetic cardiomyopathy: restoration of microvascular homeostasis and recovery of cardiac function in diabetic cardiomyopathy after replenishment of local vascular endothelial growth factor. Circulation.

[B8] Broderick TL, Kopp SJ, Daar JT, Romano FD, Paulson DJ (1994). Relation of glycosylated hemoglobin to in vivo cardiac function in response to dobutamine in spontaneously diabetic BB Wor rats. Can J Physiol Pharmacol.

[B9] Fazan R, Ballejo G, Salgado MC, Moraes MF, Salgado HC (1997). Heart rate variability and baroreceptor function in chronic diabetic rats. Hypertension.

[B10] Fazan R, Dias da Silva VJ, Ballejo G, Salgado HC (1999). Power spectra of arterial pressure and heart rate in streptozotocin-induced diabetes in rats. J Hypertens.

[B11] Elhendy A, Tsutsui JM, O'Leary EL, Xie F, McGrain AC, Porter TR (2005). Noninvasive diagnosis of coronary artery disease in patients with diabetes by dobutamine stress real-time myocardial contrast perfusion imaging. Diabetes Care.

[B12] Fein FS, Strobeck JE, Malhotra A, Scheuer J, Sonnenblick EH (1981). Reversibility of diabetic cardiomyopathy with insulin in rats. Circ Res.

[B13] Schaan BD, Maeda CY, Timm HB, Medeiros S, Moraes RS, Ferlin E, Fernandes TG, Ribeiro JP, Schmid H, Irigoyen MC (1997). Time course of changes in heart rate and blood pressure variability in streptozotocin-induced diabetic rats treated with insulin. Braz J Med Biol Res.

[B14] Pollack PS, Malhotra A, Fein FS, Scheuer J (1986). Effects of diabetes on cardiac contractile proteins in rabbits and reversal with insulin. Am J Physiol.

[B15] Stroedter D, Schmidt T, Bretzel RG, Federlin K (1995). Glucose metabolism and left ventricular dysfunction are normalized by insulin and islet transplantation in mild diabetes in the rat. Acta Diabetol.

[B16] Villanueva DS, Poirier P, Standley PR, Broderick TL (2003). Prevention of ischemic heart failure by exercise in spontaneously diabetic BB Wor rats subjected to insulin withdrawal. Metabolism.

[B17] Ramanadaham S, Tenner TE (2006). Chronic effects of streptozotocin diabetes on myocardial sensitivity in the rat. Diabetologia.

[B18] Kusaka M, Kishi K, Sokabe H (1987). Does so-called streptozocin hypertension exist in rats?. Hypertension.

[B19] Maeda CY, Fernandes TG, Timm HB, Irigoyen MC (1995). Autonomic dysfunction in short-term experimental diabetes. Hypertension.

[B20] Maeda CY, Fernandes TG, Lulhier F, Irigoyen MC (1995). Streptozotocin diabetes modifies arterial pressure and baroreflex sensitivity in rats. Braz J Med Biol Res.

[B21] Akiyama N, Okumura K, Watanabe Y, Hashimoto H, Ito T, Ogawa K, Satake T (1989). Altered acetylcholine and norepinephrine concentrations in diabetic rat hearts. Role of parasympathetic nervous system in diabetic cardiomyopathy. Diabetes.

[B22] Tohmeh JF, Shah SD, Cryer PE (1979). The pathogenesis of hyperadrenergic postural hypotension in diabetic patients. Am J Med.

[B23] Teshima Y, Takahashi N, Saikawa T, Hara M, Yasunaga S, Hidaka S, Sakata T (2000). Diminished expression of sarcoplasmic reticulum Ca(2+)-ATPase and ryanodine sensitive Ca(2+)Channel mRNA in streptozotocin-induced diabetic rat heart. J Mol Cell Cardiol.

[B24] Abe T, Ohga Y, Tabayashi N, Kobayashi S, Sakata S, Misawa H, Tsuji T, Kohzuki H, Suga H, Taniguchi S, Takaki M (2002). Left ventricular diastolic dysfunction in type 2 diabetes mellitus model rats. Am J Physiol Heart Circ Physiol.

[B25] Bers DM (2002). Cardiac excitation-contraction coupling. Nature.

[B26] Solaro RJ, Briggs FN (1974). Estimating the functional capabilities of sarcoplasmic reticulum in cardiac muscle. Calcium binding. Circ Res.

[B27] Bayliss LE, Muller EA, Starling EH (1928). The action of insulin and sugar on the respiratory quotient and metabolism of the heart-lung preparation. J Physiol.

[B28] Oye I, Sinclair D (1966). Insulin in myocardial infarction. Lancet.

[B29] Austin CE, Chess-Williams R (1992). Transient elevation of cardiac beta-adrenoceptor responsiveness and receptor number in the streptozotocin-diabetic rat. J Auton Pharmacol.

[B30] Uekita K, Tobise K, Onodera S (1997). Enhancement of the cardiac beta-adrenergic system at an early diabetic state in spontaneously diabetic Chinese hamsters. Jpn Circ J.

[B31] Savarese JJ, Berkowitz BA (1979). beta-Adrenergic receptor decrease in diabetic rat hearts. Life Sci.

[B32] Dinçer D, Bidasee KR, Güner S, Tay A, Özçelikay AT, Altan VM (2001). The effect of diabetes on expression of â1-, â2-, and â3-adrenoreceptors in rat hearts. Diabetes.

[B33] NICKERSON M (1956). Receptor occupancy and tissue response. Nature.

